# Elevated Serum Amyloid A Levels Contribute to Increased Platelet Adhesion in COVID-19 Patients

**DOI:** 10.3390/ijms232214243

**Published:** 2022-11-17

**Authors:** Ronen Siman-Tov, Rulla Shalabi, Amir Shlomai, Elad Goldberg, Wesam Essa, Eden Shusterman, Jacob N. Ablin, Michal Caspi, Rina Rosin-Arbesfeld, Ella H. Sklan

**Affiliations:** 1 Department of Clinical Microbiology and Immunology, The Sackler Faculty of Medicine, Tel Aviv University, Tel Aviv 6997801, Israel; 2 Department of Medicine F, Rabin Medical Center, Beilinson Hospital, Petah Tikva 4941492, Israel; 3 The Sackler Faculty of Medicine, Tel Aviv University, Tel Aviv 6997801, Israel; 4 Department of Medicine D, Rabin Medical Center, Beilinson Hospital, Petah Tikva 4941492, Israel; 5 Department of Internal Medicine H, Tel Aviv Medical Center, Tel Aviv 6423906, Israel

**Keywords:** COVID-19, platelets, SARS-CoV-2, serum amyloid A, thrombosis

## Abstract

Coronavirus disease-19 (COVID-19) patients are prone to thrombotic complications that may increase morbidity and mortality. These complications are thought to be driven by endothelial activation and tissue damage promoted by the systemic hyperinflammation associated with COVID-19. However, the exact mechanisms contributing to these complications are still unknown. To identify additional mechanisms contributing to the aberrant clotting observed in COVID-19 patients, we analyzed platelets from COVID-19 patients compared to those from controls using mass spectrometry. We identified increased serum amyloid A (SAA) levels, an acute-phase protein, on COVID-19 patients’ platelets. In addition, using an in vitro adhesion assay, we showed that healthy platelets adhered more strongly to wells coated with COVID-19 patient serum than to wells coated with control serum. Furthermore, inhibitors of integrin aIIbβ3 receptors, a mediator of platelet–SAA binding, reduced platelet adhesion to recombinant SAA and to wells coated with COVID-19 patient serum. Our results suggest that SAA may contribute to the increased platelet adhesion observed in serum from COVID-19 patients. Thus, reducing SAA levels by decreasing inflammation or inhibiting SAA platelet-binding activity might be a valid approach to abrogate COVID-19-associated thrombotic complications.

## 1. Introduction

The outcome of severe acute respiratory syndrome coronavirus 2 (SARS-CoV-2) infection ranges from asymptomatic or mild illness, in most patients, to a symptomatic, occasionally severe disease known as coronavirus disease-19 (COVID-19) [1]. Thrombotic complications, such as pulmonary embolism, strokes, and heart attack, are a hallmark of COVID-19, occurring in up to one-third of the patients’ [2,3]. Although such complications occur during other respiratory viral infections, such as influenza, the observed frequency is higher in COVID-19 patients [4]. Clinically, COVID-19 patients have elevated levels of D-dimer and other fibrin/fibrinogen degradation products. Interestingly, abnormalities in various coagulation markers, including prothrombin time, partial thromboplastin time, and platelet counts, were not observed in these patients [5]. Most hospitalized COVID-19 patients with elevated D-dimer levels receive antithrombotic therapy [6]. However, thrombi are still formed in some patients despite the thromboprophylaxis treatment [3,7]. Thrombotic complications occur in about 30% of severe COVID-19 patients but have also been described in outpatients; yet data on such patients are limited. Furthermore, the number of intensive care unit (ICU) patients developing thrombotic complications is higher than that of non-ICU patients [8].

Several mechanisms have been proposed to explain COVID-19-associated coagulopathy; most focus on the contribution of the systemic hyperinflammation associated with COVID-19, with endothelial activation and tissue damage as the underlying causes [9,10]. We hypothesized that mechanisms associated with proteomic changes in the COVID-19 patients’ platelets might contribute to the aberrant clotting observed in COVID-19 patients. Using mass spectrometry analysis of platelets from COVID-19 patients compared to controls, we identified increased levels of serum amyloid A on patients’ platelets. SAA1 and SAA2 are acute-phase proteins that share over 90% identity, whose expression is coordinately regulated and triggered by inflammation [11]. SAA is present at very low levels in the serum of healthy individuals, typically less than 3 mg/L. Serum SAA rapidly increases in the acute phase of the inflammatory response up to 1000-fold in 24 h [12].

Human platelets adhere to SAA by binding through the platelet integrin αIIβ3 receptors. SAA was suggested to modulate platelet adhesion at vascular injury sites by sharing platelet receptors with other platelet-adhesive proteins. However, its direct physiologic relevance is still unknown [13]. Here, we show that inhibitors of the integrin aIIbβ3 receptor, a mediator of platelet-SAA binding, reduced SAA binding to platelets and platelet adhesion to COVID-19 serum. These results suggest that inhibiting serum SAA levels or its binding to the integrin receptor might reduce platelet adhesion and, accordingly, COVID-19-associated thrombosis. Thus, SAA might serve as a factor linking proinflammatory and prothrombotic pathways in COVID-19 and might be a relevant factor in other pathological conditions.

## 2. Results

### 2.1. SAA Levels Are Elevated on the Platelets of COVID-19 Patients

**Figure 1:**
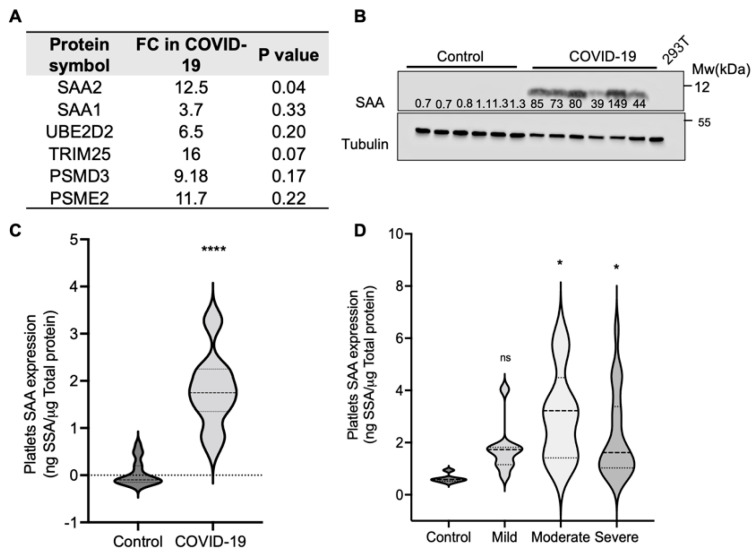
SAA is bound to platelets from COVID-19 patients. (**A**) Mass spectrometry was used to identify and quantify proteins overexpressed in purified COVID-19 platelets compared to controls. Shown are proteins whose levels were consistently elevated in two independent experiments. Values indicate the average fold-change (FC) in the relative label-free quantification intensity between the COVID-19 and the control samples (*n* = 4 in each group). Unpaired Student’s *t*-test was used to determine the significance. (**B**) Platelet lysates from control or COVID-19 donors were separated using SDS-PAGE. SAA was detected using a specific antibody. Tubulin was used as a loading control. Results are representative of six controls and six COVID-19 patients. Numbers represent fold intensity of control densitometric values normalized to tubulin. (**C**) SAA levels in platelet lysates from controls (*n* = 17) or COVID-19 patients (*n* = 15) were determined using ELISA. The dashed line in the violin plot is the median. The dotted lines show the interquartile range. Unpaired *t*-test, **** *p* < 0.001. (**D**) Platelet lysates from control or COVID-19 donors separated according to disease severity were analyzed using SDS-PAGE (*n* = 6 controls, 9 mild, 5 moderate, and 20 severe disease patients). SAA was detected using a specific antibody. The results were normalized to tubulin and to the amount of total protein. Ordinary one-way ANOVA, Dunnett’s multiple comparisons compared to control, ns = non-significant, * *p* < 0.05.

As platelets are mediators of thrombus formation, we used quantitative mass spectrometry to determine whether there are differences in the platelet protein content between COVID-19 patients and age-matched controls. Six proteins were consistently elevated by over twofold in COVID-19 patients compared to controls (Figure 1A). Among these proteins, only serum amyloid A (SAA2) was increased by 12-fold in COVID-19 patients, showing statistical significance (*p* = 0.04). SAA1, a close family member, was also elevated by 2.8-fold in COVID-19 patients, although this elevation was not statistically significant. To confirm the mass spectrometry results, we isolated platelets from COVID-19 patients and controls to test the SAA protein levels in the platelet lysates using an SAA-specific antibody (Figure 1B). The SAA levels were markedly elevated in the platelets from the COVID-19 patients, compared to the controls. While SAA is present in low amounts in the platelets of healthy individuals, our western blot analysis was not sensitive enough to detect it.

To further confirm SAA binding to platelets, we used an enzyme-linked immunoassay (ELISA) to determine the amount of SAA in COVID-19 platelet lysates vs. that in the controls (Figure 1C). SAA levels were, on average, 49.7-folds higher in COVID-19 platelet lysates than in controls, although highly variable among patients. Despite this variability, the SAA levels were significantly elevated in COVID-19 patients (*p* < 0.0001). To determine whether the SAA platelet levels correlated with COVID-19 severity, SAA levels were measured in platelet lysates from control and COVID-19 patients using western blot analysis. SAA levels in platelets were quantified by extrapolation from a known amount of recombinant SAA loaded on the same gel, normalized to tubulin, and the total protein loaded. SAA levels ranged between 0.47 and 6.33 ng SAA/μg total protein. The attending clinician determined disease severity according to the National Institute of Health guidelines [14]. The patients’ clinical data are summarized in Table 1.

The SAA levels were, on average, 3.1-fold higher in COVID-19 platelet lysates from mild patients compared to those from the controls and 4.8-fold and 4.4-fold higher in the platelets from moderate and severe patients, respectively (Figure 1D). As observed in the ELISA results, there was high variability in SAA levels between the samples in each group. However, the difference between SAA levels in the controls and in moderate and severe disease patients was statistically significant (*p* = 0.02 and *p* = 0.04). Overall, the platelet SAA levels correlated with disease severity.

### 2.2. SAA Binds COVID-19 Platelet Surface in a Dose-Dependent Manner

**Figure 2:**
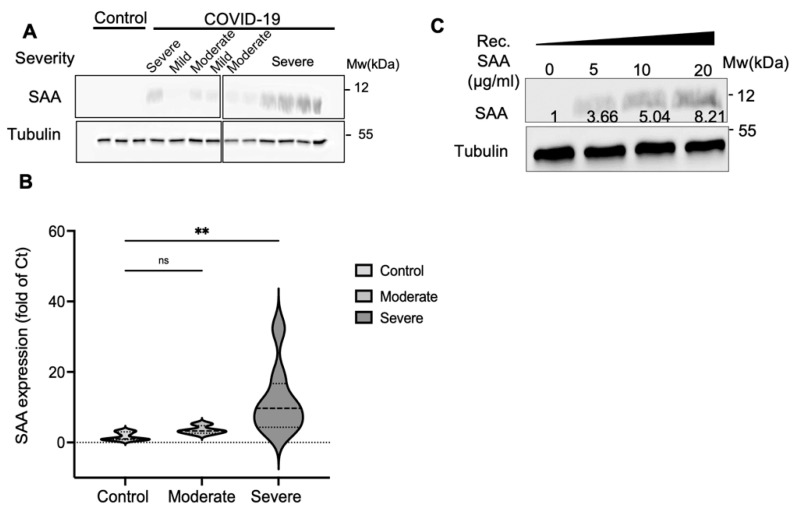
Serum SAA from COVID-19 patients binds healthy platelets in a dose-dependent manner. (**A**) Platelets from a healthy donor were incubated with serum from controls (*n* = 3) or different COVID-19 patients with varying disease severity (*n* = 10). Following incubation, the platelets were washed and lysed. SAA levels were measured by western blot. SAA was detected using a specific antibody. Tubulin was used as a loading control. (**B**) Densitometric quantification of a series of experiments performed as described in A. The results are from six independent repeats. One-way ANOVA, ns = non-significant, ** *p* < 0.01. (**C**) Platelets from a healthy donor were incubated with varying amounts of recombinant SAA for 1 h at 37 °C. The platelet protein lysates were separated using SDS-PAGE. Numbers indicate densitometric analyses of the band intensity compared to tubulin expressed as folds of the non-treated control. Shown is a representative experiment from at least three independent repeats.

To confirm that the SAA levels observed in the platelet lysates reflected the binding of serum SAA to platelets, we incubated the platelets obtained from a healthy donor in the sera from different COVID-19 patients with varying disease severity. Following incubation, the platelets were washed, lysed, and analyzed by western blotting. SAA binding levels correlated with disease severity in this assay as well (Figure 2A). The SAA binding levels were quantified by densitometry and normalized to tubulin (Figure 2B). SAA binding levels increased by 2.13-fold compared to those in the controls when serum from moderate-disease patients was used and by 7.11-fold using serum from severe-disease patients (*p* = 0.0084).

A similar experiment was performed with platelets from healthy donors incubated with recombinant SAA (5–20 μg/mL), showing that SAA bound platelets in a dose-dependent manner (Figure 2C). As average SAA levels in the serum of COVID-19 patients range from 8 to 162 μg/mL [15], the concentrations used in our experiment were well within the physiological range. Therefore, these results suggest that SAA binding to platelets reflects the patients’ serum SAA levels and correlates with COVID-19 severity.

**Figure 3:**
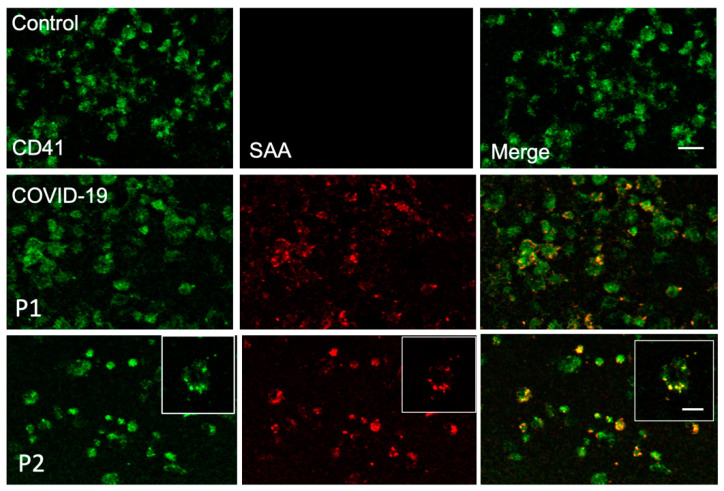
SAA partially colocalizes with CD41. Immunofluorescence was performed on washed platelets from a healthy control and COVID-19 patients (*n* = 3) using anti-SAA (red) and -CD41 (green) specific antibodies. Images were obtained using confocal microscopy. Inset: an enlarged platelet cluster showing partial colocalization. Bar = 5 µm.

To demonstrate that SAA binds to the platelet surface, immunofluorescence co-staining of SAA and CD41, an integrin αIIbβ3 subunit, was performed on platelets from COVID-19 patients and a healthy control (Figure 3). The platelets in this experiment were not permeabilized to confirm that SAA was associated with the platelet surface. SAA levels on the control platelets were set as the background value. While SAA could not be observed in the control samples, SAA partially co-localized with integrin αIIbβ3 in the patient samples.

### 2.3. COVID-19 Serum Significantly Increases Platelet Adhesion

**Figure 4:**
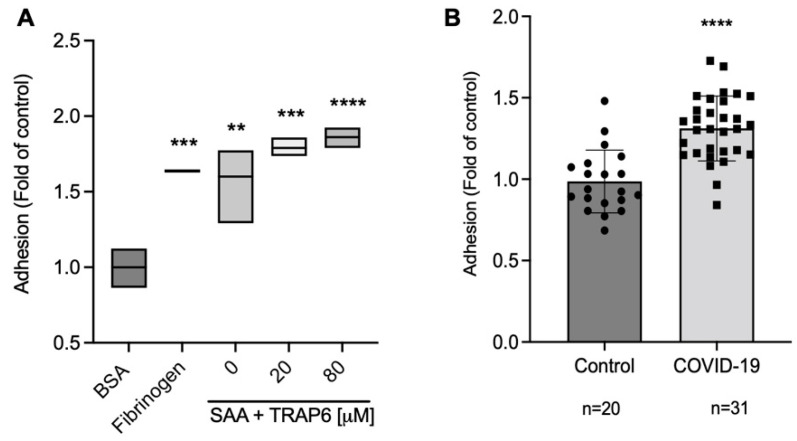
COVID-19 serum proteins increase platelet adhesion. (**A**) Adhesion of platelets to immobilized proteins. Microtiter plates were precoated with the indicated proteins in triplicates. Platelet suspensions from a healthy individual were then added to the wells. Adhesion was determined using the acid phosphatase assay. BSA was used as a negative control for background adhesion. Recombinant SAA and fibrinogen served as positive controls. TRAP-6, a platelet activator, was used to increase SAA binding in the indicated wells. Values are the mean ± standard deviation from three independent experiments. Ordinary one-way ANOVA, Dunnett’s multiple comparisons test, ** *p* < 0.002, *** *p* < 0.0002, **** *p* < 0.0001. (**B**) Adhesion of platelets to COVID-19 patient serum. Microtiter plates were precoated overnight with COVID-19 (*n* = 31) or control serum (patients with non-inflammatory diseases or healthy individuals, *n* = 20). Platelet suspensions from healthy individuals were added to the wells. Adhesion was determined using the acid phosphatase assay. Unpaired Student’s *t*-test, ****, *p* < 0.0001.

Platelets were previously shown to adhere to SAA [13]. Thus, we tested if proteins from the serum of COVID-19 patients could increase the adhesion of platelets from healthy individuals using a microtiter plate adhesion assay. To confirm the efficiency of our assay, we tested the adhesion of platelets to immobilized recombinant SAA (Figure 4A). Bovine serum albumin (BSA) was used as a negative control for background adhesion. In contrast, fibrinogen was used as a positive control as described [13]. Both fibrinogen (1.63-fold, *p* = 0.0008) and SAA (1.6-fold, *p* = 0.0013) significantly increased the adhesion levels compared to BSA. To increase the range of the assay, thrombin receptor-activating peptide-6 (TRAP-6), a platelet activator, was added to some of the SAA-coated wells [13]. The resulting activation did not significantly differ from that measured with SAA alone. Thus, TRAP-6 was not used in the rest of the experiments.

To test the role of factors within COVID-19 patient serum in platelet adhesion, we coated the microtiter plate wells with patients’ serum (*n* = 31), using the serum of patients with non-inflammatory diseases or of healthy individuals as a control (*n* = 20). Adhesion was tested using platelets from healthy individuals, and as shown in Figure 4B, platelet adhesion was significantly increased in the presence of COVID-19 patient serum compared to that observed with control serum (1.31-folds, *p* < 0.0001). Of note, all the samples used in our study were serum samples devoid of fibrinogen and other clotting factors, strongly suggesting that COVID-19 serum contains other factors contributing to increased platelet adhesion.

### 2.4. COVID-19 Serum SAA Promotes Adhesion by Binding to Platelet Integrin αIIβ3 Receptors

Due to the large amounts of SAA in the serum of COVID-19 patients, attempts to outcompete SAA and prevent its binding to platelets or reduce platelet adhesion using an anti-SAA antibody or SAA-derived peptides were unsuccessful. In addition, attempts to deplete SAA from COVID-19 serum using immunoprecipitation with large amounts of anti-SAA antibodies were also ineffective (not shown).

**Figure 5:**
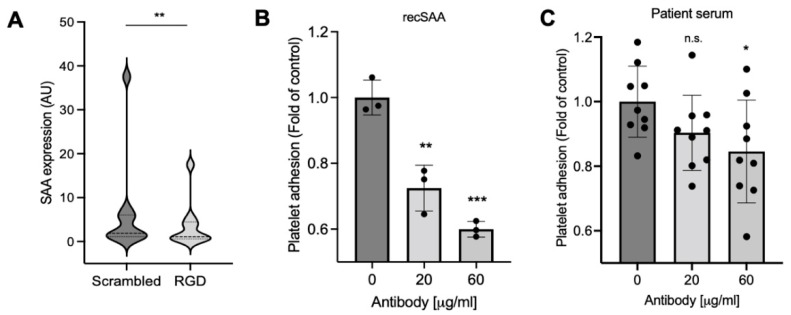
SAA increases platelet adhesion through the integrin receptor. (**A**) Platelets from a healthy donor were incubated at 37 °C for 1 h with COVID-19 serum with an RGD or a scrambled (Scr) peptide. The platelet protein lysates were separated using SDS-PAGE. SAA was detected using a specific antibody. Tubulin was used as a loading control. Densitometric results are shown using the serum from 10 patients. Wilcoxon test, ** *p* = 0.004. (**B**) Recombinant SAA was used to coat the wells of a microtiter plate. Healthy platelets were incubated with or without a specific anti-integrin αIIβ3 receptor antibody. The treated platelets were washed and added to the SAA-coated wells. Adhesion was determined using the acid phosphatase assay. (**C**) Healthy platelets were incubated with or without anti-integrin αIIβ3 receptor-specific antibodies. The treated platelets were washed and added to wells coated with COVID-19 patient serum. Adhesion was determined using the acid phosphatase assay. Values are the mean ± standard deviation from three independent experiments. Ordinary one-way ANOVA, Dunnett’s multiple comparisons test, ns = non-significant, * *p* < 0.05, ** *p* < 0.01, *** *p* < 0.001.

SAA was previously shown to mediate platelet adhesion by binding to the platelet integrin αIIβ3 receptors [13]. Thus, to overcome the excess amounts of SAA in COVID-19 serum, we attempted to block its receptor. RGD is an integrin recognition motif that acts as an inhibitor of integrin-ligand interactions [16]. To show that SAA-mediated binding to the integrin receptor mediates the increased adhesion observed in COVID-19 patient serum, platelets from a healthy donor were incubated with the serum from COVID-19 patients in the presence of an RGD peptide or a scrambled control (Figure 5A). The platelets were washed, and SAA binding was detected by western blot. The RGD peptide significantly inhibited SAA binding by an average of 37% (*p* = 0.0003). To further confirm that the increased adhesion was integrin-dependent, we incubated healthy platelets with a specific antibody against integrin αIIβ3 and tested their adhesion to immobilized recombinant SAA (Figure 5B). The adhesion levels were significantly reduced by 24.6% with 20 µg/mL of antibody (*p* = 0.0018) and by 38% with 60 µg/mL of antibody (*p* = 0.0002). Similar but more variable results indicating a 15.4% reduction with 60 µg/mL of antibody were obtained when the source of SAA was patients’ serum (Figure 5C, *p* = 0.035). These results suggest that at least part of the increased adhesion observed for platelets incubated with COVID-19 serum is mediated by SAA binding to platelet integrin receptors. 

## 3. Discussion

The mechanisms underlying thrombotic complications in COVID-19 patients have been extensively studied since the beginning of the COVID-19 pandemic (see [17,18,19,20,21] for recent reviews). Here, we examined whether platelets isolated from COVID-19 patients bind specific circulating proteins, contributing to the increased thrombosis. Our results show that SAA binds to platelets in COVID-19 patients and that SAA binding to platelets correlates with disease severity. While direct SAA binding to COVID-19 patients’ platelets was never tested, these results agree with previous studies showing that serum SAA levels can be used as an inflammation marker and as prognostic markers reflecting COVID-19 severity [15,22,23,24,25,26]. To verify that SAA binding to platelets demonstrates the binding of serum SAA, we incubated patients’ serum with platelets from healthy donors. Indeed, the healthy platelets bound the serum SAA, and the binding levels correlated with disease severity. A similar experiment with recombinant SAA confirmed that SAA binds platelets in a dose-dependent manner.

Platelets were previously shown to adhere to immobilized SAA. While the physiological significance of this binding is still unknown, it was hypothesized that SAA might have a role in modulating platelet adhesion at vascular injury sites [13]. Accordingly, using a platelet microplate adhesion assay, we showed increased platelet adhesion to wells coated with COVID-19 patient serum, indicating that a factor(s) in this serum contribute(s) to the elevated adhesion levels. SAA-induced adhesion was previously shown to be inhibited by the peptide GRGDSP, an inhibitor of integrin–ligand interactions. Furthermore, the integrin receptor aIIbβ3 seems to function as the platelet receptor for SAA, and its inhibition prevents platelet binding to SAA [13]. In line with these findings, incubating platelets with an RGD peptide inhibited their binding to recombinant SAA. In addition, incubation of healthy platelets with an antibody specific for the integrin receptor aIIbβ3 inhibited their binding to wells coated with recombinant SAA or with COVID-19 patient serum. These results suggest that SAA might be one of the factors contributing to the increased adhesion observed in COVID-19 serum.

Integrin αIIbβ3 is also known to bind other adhesion-related RGD-containing ligands, including its major ligand fibrinogen, but also fibrin, von Willebrand factor (vWF), and fibronectin [27]. However, all the samples used in our study were serum samples. Thus, they did not contain fibrinogen or other factors, suggesting that COVID-19 serum contains other factors contributing to the observed elevated adhesion. 

The hypothesis that the overproduction of SAA can cause systemic amyloidosis, i.e., amyloid deposits in various organs that promote thrombosis, together with our findings described above, led us to explore further the possible role of SAA in COVID-19-associated increased adhesion [28,29]. This notion is further supported by an additional study showing that the presence of SAA increased the amyloid formation by fibrinogen and promoted a platelet prothrombotic state [30]. These results were further confirmed in our hands, where recombinant SAA incubated with healthy platelets significantly increased their binding to fibrinogen by 10% compared to that observed with untreated platelets (not shown). 

Several additional biological functions attributed to SAA were previously published (summarized in [31]). SAA was previously shown to inhibit thrombin-induced platelet activation and aggregation but not activation induced by collagen or adenosine diphosphate [32]. These results were obtained with purified SAA with limited solubility. Furthermore, these findings were only observed with purified platelets and were not reproduced under more physiological conditions, such as with platelet-rich plasma. In our system, platelet activation by increasing concentrations of thrombin receptor-activating peptide-6 (TRAP-6) in the presence of SAA did not significantly elevate platelet adhesion compared to that observed with SAA alone (Figure 4), suggesting platelet activation does not significantly affected our findings. Furthermore, SAA was previously reported to affect leukocyte adhesion [33,34,35,36,37]. Thus, future experiments should also address the role of elevated SAA levels in COVID-19 patients in leukocyte adhesion or platelet–leukocyte interactions.

Recombinant SAA was used as a control in some of our experiments. However, these results should be interpreted with caution as some commercial recombinant SAA preparations were shown to be contaminate with bacterial components altering their biological activities [31]. An additional limitation of our study is that our initial mass spectrometry analysis was performed on a relatively small number of samples (four controls and four COVID-19 patients). A larger sample size might have identified additional contributing factors. COVID-19 severity and outcome are associated with existing co-morbidities [38]. Thus, a control group with similar co-morbidities should be used when comparing COVID-19 patients with controls. Except for asthma and COPD, which were absent in the control group, co-morbidities were mainly similar in both groups (Table S1). While asthma and COPD were in relatively low abundance and might not be significant to thrombosis, this is a notable limitation of our study. 

While further studies are necessary to establish the role of SAA in platelet adhesion, our results suggest that inhibition of SAA binding to platelets or reduction of serum SAA levels might decrease COVID-19-associated thrombotic complications. Our experimental attempts to inhibit SAA binding to platelets using competition or anti-SAA antibodies were unsuccessful, most likely due to the large amounts of SAA detected in the COVID-19 patient sera. Furthermore, an approved efficient inhibitor of SAA is unavailable, and there are no commercially available effective reagents to achieve this goal. Thus, a significant limitation of our study is that we could not provide experimental in vivo proof that SAA reduction would resolve COVID-19-mediated thrombosis. Alternative approaches might include the use of drugs that block the SAA integrin receptor aIIbβ3, such as abciximab, eptifibatide, and tirofiban [39], or the use of inhibitors of proteins implicated in the inflammatory response that were previously shown to reduce the SAA levels [40]. Both approaches were attempted by others and were shown to be efficient in lowering COVID-19 hypercoagulation [41,42,43]. These approaches, however, are indirect and affect other pathways that can independently reduce inflammation and thrombosis. Last, COVID-19-associated hypercoagulation is most likely driven by a multi-factorial process [9,10]. Our data add SAA to the factors contributing to this process, mediating an undervalued important complication of COVID-19. Further studies are warranted to assess the magnitude of this contribution.

## 4. Materials and Methods

### 4.1. Proteins, Antibodies, and Kits

Human Serum Amyloid A1 DuoSet ELISA was from R&D systems (DY3019-05). Recombinant Human Serum Amyloid A1 was from Novus Biologicals (NBP2-34875). Recombinant Human TRAP6 was from Tocris (cat #3497). Fibrinogen from human plasma (cat #F3879) and anti-Tubulin antibody (1:10,000), prostaglandin E1 (P5515), p-Nitrophenyl Phosphate (487663) were all purchased from Sigma-Aldrich Israel. Anti-Serum Amyloid A antibody was from Abcam (1:3000; cat # ab687), Anti-Integrin αIIbβ3 was from Santa Cruz (sc-21783), and Anti-CD41 was from ThermoFisher (cat # 11-0411-82). Anti-mouse and anti-rabbit secondary antibodies were obtained from Jackson ImmunoResearch (1:10,000). The RGD-containing peptide GRGDSP and scrambled control peptide GRGESP were synthesized at the Blavantik center for drug discovery at Tel Aviv University. 

### 4.2. Study Design and Population 

Hospitalized adults (≥18 years of age, Rabin Medical Center, Israel) with mild, moderate, or severe COVID-19 (national institute of health guidelines [14]) were selected for this study. The controls were hospitalized adults with non-inflammatory conditions or healthy individuals (*n* = 34 COVID-19 patients and 20 controls). The demographic and clinical parameters were obtained from the patients’ records (Table 1 and Table S1). This study was approved by the RMC institutional review board (# 0332-20 RMC) and the Tel Aviv university ethics committee (0002434-2).

### 4.3. General Reagents and Buffers

The following buffers were used for platelet purification. The platelet wash buffer contained 10 mM sodium citrate, 150 mM NaCl, 1 mM EDTA, 1% (*w*/*v*) dextrose, pH 7.4. The HEP buffer contained 140 mM NaCl, 2.7 mM KCl, 3.8 mM HEPES, 5 mM EGTA, pH 7.4. The Tyrode buffer was composed of 134 mM NaCl, 12 mM NaHCO_3_, 2.9 mM KCl, 0.34 mM Na_2_HPO_4_, 1 mM MgCl_2_, 10 mM HEPES, pH 7.4. The platelet lysis buffer 2× was composed of 2% NP40, 30 mM HEPES, 150 mM NaCl, 2 mM EDTA, pH 7.4. The acid phosphatase solution contained 0.1 M citrate buffer pH 5.4, containing 5 mM p-nitrophenyl phosphate and 0.1% Triton X-100.

### 4.4. Blood Fractionation

Fresh whole blood was collected into K3EDTA tubes, mixed gently, and centrifuged at 200× *g* for 20 min at room temperature without brakes. To purify the platelets, platelet-rich plasma (PRP) was resuspended with HEP buffer (including 1 µM prostaglandin E1 to prevent platelet activation) at a 1:1 ratio (*v*/*v*), then mixed gently and spun at 800× *g* for 20 min at room temperature. The supernatants were discarded, and the packed platelets were washed carefully two times by adding the platelet wash buffer without resuspension. Before the analysis, platelet concentration was adjusted as needed with Tyrode buffer. For platelet protein lysates, an ice-cold 1:1 mixture of Tyrode buffer and 2× platelet lysis buffer was used, containing a protease inhibitor cocktail (Sigma). The cells were centrifuged at maximal speed for 15 min at 4 °C following incubation for 20 min on ice. For serum collection, blood was drawn into a serum separation tube, mixed gently, and centrifuged at 200× *g* for 20 min at room temperature without brakes. The serum was kept at −80° until analysis.

### 4.5. In-Gel Proteolysis and Mass Spectrometry Analysis

Mass spectrometry was used to identify and quantify proteins overexpressed in purified COVID-19 platelets compared to the controls. The proteins in the gel were reduced with 2.8 mM DTT (60 °C for 30 min), modified with 8.8 mM iodoacetamide in 100 mM ammonium bicarbonate (in the dark, at room temperature for 30 min), and digested in 10% acetonitrile and 10 mM ammonium bicarbonate with modified trypsin (Promega) at a 1:10 enzyme-to-substrate ratio, overnight at 37 °C. Additional trypsinization was conducted for 4 h. The resulting tryptic peptides were resolved by reverse-phase chromatography on 0.075 × 200 mm fused silica capillaries (J&W) packed with Reprosil reversed-phase material (Dr. Maisch GmbH, Germany). The peptides were eluted with a linear 105 min gradient of 5% to 28% acetonitrile with 0.1% formic acid in water, a 15 min gradient of 28% to 90% acetonitrile with 0.1% formic acid in water, and then for 15 min with 90% acetonitrile with 0.1% formic acid in water at flow rates of 0.15 μL/min. Mass spectrometry was performed by a Q-Exactive plus mass spectrometer (QE, Thermo, MA USA) in a positive mode using a repetitively full MS scan followed by high-energy collision dissociation (HCD) of the 10 most dominant ions selected from the first MS scan. The mass spectrometry data were analyzed using the MaxQuant software 1.5.2.8 [44] for peak picking and identification using the Andromeda search engine, searching against the human proteome from the Uniprot database with a mass tolerance of 6 ppm for the precursor masses and 20 ppm for the fragment ions. Oxidation on methionine and protein N-terminus acetylation were accepted as variable modifications, and carbamidomethyl on cysteine was accepted as a static modification. The minimal peptide length was set to six amino acids, and a maximum of two miscleavages was allowed. The data were quantified by label-free analysis using the same software. Peptide- and protein-level false discovery rates (FDRs) were filtered to 1% using the target decoy strategy. The protein tables were filtered to eliminate identifications from the reverse database, common contaminants, and single peptide identifications. Mass spectrometry analysis was performed at the Smoler Proteomics Center at the Technion.

For the statistical analysis, we combined two sets of samples sent independently for mass spectrometry. In each set, we had two samples in each group (i.e., samples from two controls and two COVID-19 patients); thus, each group contained four samples.

### 4.6. SDS-PAGE and Western Blot Analysis

The concentrations of the platelet lysates were measured using the Bradford assay, according to the manufacturer’s instructions. The lysates were loaded onto a 12.5% SDS-polyacrylamide gel. Following electrophoretic separation, proteins were transferred to nitrocellulose membranes and blocked with 5% low-fat milk. The membranes were incubated with specific anti-SAA and anti-tubulin primary antibodies, washed with PBS containing 0.001% Tween-20 (PBST), and incubated with the appropriate horseradish peroxidase-conjugated secondary antibody. After washing in PBST, the membranes were subjected to enhanced chemiluminescence (ECL) detection. Quantification of band intensity was performed by ImageJ software version 1.53 or FusionCapt Advance software version 16.

### 4.7. Immunofluorescence 

Washed platelets were allowed to adhere to glass coverslips pre-coated with poly-L-lysine (Sigma, Rehovot, Israel, P4832) for 1 h at room temperature. The cells were washed twice with PBS and fixed with 1% paraformaldehyde. The cells were blocked with 2% BSA for 1 h and then incubated with a mouse monoclonal anti-SAA antibody (sc-59679) for an additional hour. The coverslips were co-stained with Alexa Fluor 594 Phalloidin (Thermo, MA, USA, A12381) and an Alexa-Fluor 488 secondary antibody. Images were obtained using confocal microscopy (LSM800, Carl Zeiss, Oberkochen, Germany) with a ×60 oil-immersed objective lens.

### 4.8. Platelet Adhesion

The platelet adhesion assay was performed as previously described [13,45]. Microtiter plates were precoated overnight at 4 °C with 100 µL of the adhesive protein solutions: BSA (50 μg/mL), fibrinogen (50 μg/mL), SAA (2 μg/mL), or COVID-19 or control (patients with non-inflammatory diseases or healthy individuals) human serum. TRAP-6 (20 μM), a platelet activator, was used to increase SAA binding in the indicated wells. The wells were then blocked with Tyrode buffer containing 1% BSA for 1 h at 37 °C. The platelets were allowed to adhere by adding a 10^5^ platelet suspension to the precoated wells for 1 h at 37 °C. After the wells were washed carefully with Tyrode buffer to remove unattached platelets, acid phosphatase was used to quantify the number of adherent platelets. The acid phosphatase solution (150 µL) was added to each well for 1 h at room temperature. The reaction was stopped by adding 100 µL of 2 N NaOH. Finally, the absorbance was measured at 405 nm using a microplate reader. To test the effect of various agents on platelet adhesion, platelets were preincubated for 30 min at 37 °C with the indicated agent before being added to the precoated wells. All experiments were performed in at least triplicates.

### 4.9. ELISA

An SAA-specific ELISA (SAA1 DuoSet ELISA Kit, R&D Systems, MN USA) was used to detect and quantify the SAA levels in the platelets of healthy and COVID-19 patients according to the manufacturer’s instructions. All steps were performed at room temperature. Briefly, microtiter plates were coated overnight with the capture antibody and blocked with a reagent diluent for 1 h. Then, 100 µL of a solution containing 5 µg of platelet total protein lysate was incubated with the capture antibody for 2 h, followed by the detection antibody for 2 h and the horseradish peroxidase and substrate solution for an additional 20 min. Finally, the reaction was stopped using a stop solution, and the absorbance was measured using a microplate reader at a wavelength of 540 nm and with a second reading at 450 nm to account for the plate background. The SAA amounts were determined according to the kit standards and divided by the amount of total protein loaded.

### 4.10. Statistical Methods

Data were analyzed using Graphpad Prism software (version 9.4.1, GraphPad, La Jolla, CA, USA). The results are presented as the mean with standard deviation of 3–6 independent experiments, as indicated in the legend section. An unpaired t-test or analysis of variance (ANOVA) was used to assess the significance of the variations; multiple comparisons were conducted according to software recommendations.

## Figures and Tables

**Table 1:** Clinical and demographic characteristics of the patients who participated in the study.

	Control (*n* = 20)	COVID-19 (*n* = 34)	*p* Value (*t* Test)
Gender male %	68.4	56	
Age (median, IQR)	59 (49–65.5)	64.5 (51.2–74)	0.19
Severe %		59	
Moderate %		20.5	
Mild %		20.5	
ddimer max mg/L (median, IQR)		805 (632–1530)	
CRP max mg/dL (median, IQR)	5.5 (1.6–11.46)	18.8 (10.9–24.6)	0.01
Platelets min k/μL(median, IQR)	235 (208–283)	171.5 (108.3–207.3)	0.006
Platelets max k/μL (median, IQR)	327 (268–363)	363 (245–473)	0.57
Fibrinogen max mg/dL (median, IQR)	731 (514–953.3)	782 (704.5–871.8)	0.49
Troponin ng/L (median, IQR)	14 (13–37.5)	17 (13–45.79)	0.35
Creatinine high mg/dL (median, IQR)	0.98 (0.81–1.15)	1.1 (0.77–1.92)	0.19
INR min (median, IQR)	0.99 (0.95–1.1)	1.12 (1.04–1.2)	0.29
INR max (median, IQR)	1.28 (1.13–1.36)	1.3 (1.2–1.53)	0.58
Antiplatelet %	31.5	14.7	
Lactate max mg/dL (median, IQR)	22.5 (21.25–23.7)	26 (21.5–32.5)	0.3

## Data Availability

Not applicable.

## Supplementary Materials

The supporting information can be downloaded at: https://www.mdpi.com/article/10.3390/ijms232214243/s1.
