# Supplementary material for: Elevated Serum Amyloid A Levels Contribute to Increased Platelet Adhesion in COVID-19 Patients
Source: Int J Mol Sci. 2022 Nov 17;23(22):14243. doi: 10.3390/ijms232214243 (PMC9692251; doi:10.3390/ijms232214243)
Supplement: Supplementary file 1 [file ijms-23-14243-s001.zip › ijms-1956525-supplementary.pdf]

Table S1. Comorbidities of the patients that participated in the study. List of comorbidities expressed as a percent.

	Controls	COVID-19
**Hypertension**	29.6	38.2
**Cardiac**	14.8	26.5
**Diabetes mellitus**	22.2	35.3
**Astma**	0	11.8
**COPD**	0	5.9
**Metabolic**	18.5	29.4
**Solid cancer**	7.4	8.8
**Renal**	3.7	8.8
**Gastrointestinal**	3.7	2.9
**Obesity**	3.7	8.8
